# Effects of Liposome‐Encapsulated α‐Pinene on In Vitro Oocyte Maturation and Embryo Development in Bovine Species

**DOI:** 10.1002/mrd.70094

**Published:** 2026-02-19

**Authors:** Venância Antonia Nunes Azevedo, Maria Alice de Almeida, Matheus de Andrade Chaves, Luca Angi Souza, Luiziana Cavalcante Costa Fernandes Crisóstomo, Mariana Aragão Matos Donato, Christina Alves Peixoto, Josimar Oliveira Eloy, Flávio Vieira Meirelles, Felipe Perecin, Juliano Coelho da Silveira, José Roberto Viana Silva

**Affiliations:** ^1^ Laboratory of Biotechnology and Physiology of Reproduction Federal University of Ceara Sobral Brazil; ^2^ Department of Veterinary Medicine, Faculty of Animal Science and Food Engineering University of São Paulo Pirassununga Brazil; ^3^ School of Pharmacy, Odontology and Nursing Federal University of Ceara Fortaleza Brazil; ^4^ Laboratory of Ultrastructure, CPqAM/FIOCRUZ Federal University of Pernambuco Recife Brazil

**Keywords:** antioxidant activity, In vitro embryo production, liposomal encapsulation, oocyte maturation, α‐pinene

## Abstract

Oocyte in vitro maturation (IVM) represents a crucial phase in embryo production, often compromised by reactive oxygen species (ROS). Strategies to mitigate ROS are essential to improve oocyte quality. This study investigated the effects of liposome‐encapsulated α‐pinene (Lip‐α‐pinene) on bovine oocyte IVM and embryonic development following parthenogenetic activation. Cumulus‐oocyte complexes (COCs) were matured in vitro for 22–24 h in control medium or supplemented with Lip‐blank, or 0.01, 1.0 or 100.0 µg/mL Lip‐α‐pinene. Lip‐α‐pinene had a size of 75.86 ± 0.95 nm, low polydispersity (0.26 ± 0.00), and zeta potential of −31.55 ± 2.23 mV. Nuclear maturation was not affected. However, COCs matured with 1.0 µg/mL Lip‐α‐pinene showed well‐preserved ultrastructure of oocyte and cumulus cells, reduced ROS levels and lipid accumulation compared to control, Lip‐blank, and 100.0 µg/mL Lip‐α‐pinene. This was accompanied by relative abundance of *NRF2*, *SOD*, and *PRDX6*. Furthermore, 1.0 and 100.0 µg/mL Lip‐α‐pinene increased cleavage rates and cells per blastocyst compared to control, while blastocyst rates and lipid content were not affected. In conclusion, 1.0 µg/mL Lip‐α‐pinene enhances antioxidant capacity of bovine oocytes by reducing ROS and lipid accumulation, associated with abundance of *NRF2*, *SOD*, and *PRDX6* transcripts, thereby improving oocyte competence and quality of parthenogenetic embryos.

**Abbreviations**
AFM: atomic force microscopy
ATF6: transcription factor 6
B: blastocyst
CAT: catalase
CCs: cumulus cells
COCs: cumulus‐oocyte complexes
DLS: dynamic light scattering
eB: expanded blastocyst
EE: encapsulation efficiency
ER: endoplasmic reticulum
GAPDH: glyceraldehyde‐3‐phosphate dehydrogenase
GPX1: glutathione peroxidase 1
GSH: glutathione
hB: hatched blastocyst
HPLC: high‐performance liquid chromatography
iB: initial blastocyst
IRE: inositol requiring enzyme‐1
IVM: in vitro maturation
KEAP1: kelch‐like ECH‐associated protein 1
lip‐blank: empty liposomes
Lip‐α‐pinene: liposome‐encapsulated α‐pinene
NRF2: nuclear factor erythroid 2‐related factor 2
NTA: nanoparticle tracking analysis
PDI: polydispersity index
PERK: kinase RNA‐like endoplasmic reticulum kinase
PRDX6: peroxiredoxin 6
ROS: reactive oxygen species
SOD: superoxide dismutase
ZP: zeta potential

## 1 Introduction

In vitro maturation (IVM) of oocytes and subsequent embryo production play a pivotal role in advancing livestock reproduction, enhancing production efficiency, reducing the generation interval, and accelerating the genetic gain (Davoodian et al. 2024; Alkan et al. 2025). According to Viana (2024), the global number of embryos produced in vitro is steadily increasing; however, the developmental competence of oocytes matured in vitro remains inferior to that of those matured in vivo under physiological conditions (Lu et al. 2025). Oocytes inherently store thousands of maternal RNAs and proteins essential for early embryonic development (Cheng et al. 2022), yet the in vitro culture conditions used during IVM require further optimization to support these crucial molecular components.

During IVM, oocytes are exposed to suboptimal conditions including elevated O₂ concentrations, ultraviolet light, and fluctuations in temperature and pH. These factors contribute to an excessive accumulation of reactive oxygen species (ROS), while the absence of maternal control systems diminishes the activity of antioxidant enzymes (Keane and Ealy 2024). Elevated ROS levels compromise oocyte competence by oxidizing RNA, DNA, and proteins, disrupting cellular integrity through lipid peroxidation, and inducing mitochondrial dysfunction, endoplasmic reticulum stress, lipid accumulation, and altered gene expression patterns (Amin et al. 2014). Consequently, the supplementation of IVM media with antioxidants has been proposed as a strategy to counteract oxidative stress and improve oocyte quality (Silva et al. 2022; Keane and Ealy 2024; Lu et al. 2025).

The α‐pinene, the most abundant naturally occurring monoterpene, has garnered significant interest in biomedical research due to its diverse biological activities, particularly its antioxidant and cytoprotective effects (Porres‐Martínez et al. 2016; Karthikeyan et al. 2018; Xanthis et al. 2021; Allenspach and Steuer 2021). Studies have demonstrated that α‐pinene reduced ROS generation, lipid peroxidation, and DNA damage in HaCaT cells exposed to ultraviolet‐A radiation (320–400 nm) (Karthikeyan et al. 2018). Furthermore, α‐pinene has been shown to upregulate the expression of antioxidant defense‐related enzymes in bovine ovarian tissue, thereby enhancing follicular survival (Azevedo et al. 2024). However, its hydrophobicity, high photosensitivity, and volatility pose significant challenges to its bioefficacy and limit its direct application in biological systems (Neeman et al. 2017). To overcome these limitations, the encapsulation of α‐pinene in nanocarriers, such as liposomes offers a promising solution, enhancing its stability, bioavailability, and cellular uptake (Hammoud et al. 2021; Gulzar et al. 2024). Liposomes are particularly suitable for this purpose due to their phospholipid bilayer structure, which closely resembles that of cellular membranes, facilitating efficient delivery and protection of encapsulated compounds in biological systems, a feature that is especially attractive for application during oocyte IVM. However, to the best of our knowledge, the effect of α‐pinene during in vitro maturation of bovine oocytes are still not understood, especially in the context of its encapsulated use.

Giving the critical role of oocyte maturation in the success of in vitro embryo production, we hypothesized that the liposomal delivery of α‐pinene during IVM would enhance antioxidant defenses, reduce ROS levels, and ultimately improve oocyte quality and embryonic developmental outcomes following parthenogenetic activation. Accordingly, the objectives of this study were: (i) to formulate liposomes using the lipid film hydration technique and (ii) to evaluate their effects on oocyte maturation. The parameters assessed included: (a) nuclear and cytoplasmic maturation, (b) intracellular ROS and glutathione (GSH) levels, (c) oocyte and cumulus cells ultrastructure, (d) developmental competence to the blastocyst stage, (e) lipid content in matured oocytes and blastocysts, and (f) expression levels of mRNAs associated with oxidative stress and endoplasmic reticulum (ER) stress in matured oocytes.

## 2 Material and Methods

### 2.1 Chemicals and Reagents

Unless otherwise indicated, all reagents were purchased from Sigma‐Aldrich (St. Louis, MO, USA).

### 2.2 Production of Liposomes

Liposomal formulations containing α‐pinene (Lip‐α‐pinene) were prepared using the thin lipid film hydration method, as originally described by Bangham et al. (1965). Briefly, the lipid phase was composed of soybean phosphatidylcholine (50 mg) (Lipoid, Ludwigshafen, Germany, No. 97281‐47‐5) and cholesterol (2.48 mg), in a molar ratio 10:1. The lipids and α‐pinene were mixed at a 1:10 ratio and dissolved in 2 mL of chloroform. The organic solvent was removed using a rotary evaporator (IKA, model RV8) under reduced pressure at 100 rpm resulting in the formation of a thin dry lipid film after 30 min. The film was subsequently hydrated with 10 mL of PBS (pH 7.4) at 60°C for 30 min at 100 rpm. To obtain uniformly sized liposomes, the dispersion was sonicated using a probe‐type ultrasonicator (Q500; QSonica, Newtown, USA) at 20% amplitude, employing 5/2 on/off pulse cycles for 7.5 min in an ice bath, as described by Eloy et al. (2016). Blank liposomes (Lip‐blank) were prepared following the same procedure, excluding α‐pinene.

### 2.3 Physicochemical Characterization of Liposomes

#### 2.3.1 Particle Size, Polydispersity and Zeta Potential

The mean particle size and polydispersity index (PDI) of the liposomes were determined via dynamic light scattering (DLS) using a Zetasizer Nano ZS (ZEN3600, Malvern Instruments, Worchester, UK). Zeta potential (ZP) was measured by electrophoretic light scattering, using the same instrument. For all measurements. Liposomal formulations were diluted 1:10 in ultrapure water prior to analysis, as previously described by Eloy et al. (2020).

#### 2.3.2 Encapsulation Efficiency

Encapsulation efficiency (EE%) was assessed using a direct quantification approach. Encapsulated α‐pinene was quantified following dilution of the liposome (300 µL) sample in methanol (2.700 µL) and filtration through a 0.45 µm PTFE membrane. The resulting solution was analyzed using a validated high‐performance liquid chromatography (HPLC) method. The mobile phase consisted of water and methanol (85:15, v/v), with a flow rate of 1 mL/min through a reverse‐phase C18 column (15 cm x 4.6 mm, 3.5 µm). The injection volume was 20 µL, with detection at 204 nm and column temperature maintained at 25°C, following protocols by Eloy et al. (2020) and Hammoud et al. (2021). The method validation was done previously following RDC No. 166 of July 24, 2017. The EE% was calculated using the following equation:

$$\mathrm{EE}\left(\%\right)\;=\frac{\alpha -\mathrm{pinene concentration in the purified sample}}{\alpha -\mathrm{pinene concentration in the total sample}}\times 100\%$$

#### 2.3.3 Storage Stability

Storage stability was evaluated by monitoring changes in particle size, PDI, and ZP at 30‐day intervals over a period of 120 days at 25°C using the Zetasizer Nano ZS (ZEN3600, Malvern Instruments, Worchester, UK).

#### 2.3.4 Nanoparticle Tracking Analysis

The particle concentration and distribution size were measured using a nanoparticle tracking analysis (NTA) system (NanoSight 3000; NTA 3.1 Build 3.1.24, Malvern, UK). For this, 100 µL of each liposomal sample was first diluted in 900 µL of Milli‐Q water. Measurements were performed at 37°C by capturing five 30‐second videos per sample using an sCMOS camera (camera level 12) and a detection threshold of 5.

#### 2.3.5 Atomic Force Microscopy

The morphological characteristics of Lip‐blank and Lip‐α‐pinene were further examined by atomic force microscopy (AFM) using an NT‐MDT microscope (Solver Next, Zelenograd, Russia). Liposomal dispersions were first diluted 100‐fold in ultrapure water. A 10 µL aliquot was then deposited onto a freshly cleaved mica disc and allowed to stand for 2 min. Excess diluted liposomes was removed using filter paper when necessary. Imaging was performed in non‐contact mode with a cantilever having a force constant of 5 N/m, a resonance frequency of 150 kHz, and a scan rate of 0.7 Hz, as described by Chaves and Pinho (2020).

### 2.4 Effect of Liposome Supplementation on the Viability of Cultured Bovine Cumulus Cells

Following the retrieval of bovine cumulus‐oocyte complexes (COCs), cumulus cells (CCs) were isolated from the oocytes by enzymatic and mechanical dissociation using TrypLE™ Express Enzyme (Thermo Fisher Scientific, Waltham, Massachusetts, EUA) and repeated pipetting under the stereomicroscope. The cells were centrifuged at 500 x g for 5 min at room temperature, and the supernatant was discarded. After discarding the supernatant, the cell pellet was resuspended and cultured in α minimum essential medium (α‐MEM) supplemented with 10% fetal bovine serum (FBS) and 2% penicillin/streptomycin in 25 cm² culture flasks at 38.5°C in humidified atmosphere of 5% CO_2_. Cells were cultured for 6 days with complete medium being replaced every 2 days, once the cells reached 70%–80% confluency, they were split (i.e. passaged) and cultured for another 6 days under the same conditions. These passage 1 (P1) cells were then cryopreserved in α‐MEM: 10% FBS: Dimethyl sulfoxide (DMSO) at a ratio of 8: 1: 1. The cells were then subjected to a slow freeze surrounded by isopropanol under −80°C conditions for 24 h then moved to liquid nitrogen for storage and future culture for viability analysis. Three different cumulus cells were processed.

To assess the cytotoxicity of liposomes, CCs were seeded in 96‐well plates and treated with different concentrations of nanoparticles (Lip‐blank or Lip‐α‐pinene at 10^8^ to 10^12^ particles/mL). For Lip‐α‐pinene, this meant α‐pinene concentrations ranging from 0.01 to 100.0 µg/mL. Untreated cells served as negative controls. After 24 h of incubation at 38.5°C under 5% CO_2,_ cell viability was assessed using an MTT assay. Cells were incubated with MTT solution (5 mg/mL) for 3 h, after which the supernatant was aspirated, and 200 µL of DMSO was added to each well to solubilize the formazan crystals. Absorbance was measured at 590 nm using a FluoStar Optima microplate reader (BMG LABTECH, Ortenberg, Germany). Results were normalized to control wells and expressed as a percentage of cell viability. All assays were performed in triplicate.

### 2.5 Effects of Lip‐α‐Pinene on In Vitro Maturation and Embryo Development

Ovaries from Nellore cows (*Bos taurus indicus*) were collected at a slaughterhouse (Trabiju, São Paulo, Brazil) and transported to the laboratory in a sterile 0.9% (w/v) saline solution maintained at 35°C–37°C. To obtain COCs, antral follicles (3–6 mm in diameter) were aspirated using an 18‐gauge needle connected to a syringe. The COCs were selected in TCM‐199 medium (Gibco) modified with HEPES and supplemented with 10% FBS, 0.2 mM of pyruvate, and 50 μg/mL gentamicin sulfate (referred to as the washing medium). Only COCs exhibiting homogeneous cytoplasm and a compact, multilayered cumulus cell structure (Grades 1 and 2) were used for subsequent procedures (Ferronato et al. 2023).

A total of 3,225 COCs were subjected to in vitro maturation in groups of 40‐50. Maturation was carried out in Nunc 5‐well plates, with 500 μL medium per well, incubated at 38.5°C in a humidified atmosphere containing 5% CO_2_ for 22–24 h. The standard bovine maturation medium used was TCM‐199 (Gibco) buffered with 25 mM sodium bicarbonate and supplemented with 10% FBS, 0.2 mM sodium pyruvate, 50 µg/mL gentamicin sulfate, 0.5 µg/mL follicle‐stimulating hormone (Folltropin‐V, Bioniche Animal Health, Canada), and 5 U/mL human chorionic gonadotropin (Vetecor, Hertape Calier) (Ferronato et al. 2023). Treatments included this control medium alone, or the same medium supplemented with either empty liposomes (Lip‐blank; 10^12^ particles/mL) or Lip‐α‐pinene at concentration of 10^8^, 10^10^ and 10^12^ particles/mL (referred as 0.01, 1.0, or 100.0 µg/mL α‐pinene). These concentrations were selected based on the prior cytotoxicity assays, in which no significant impact on cumulus cell viability was observed (*p* > 0.05). This experiment was repeated 14 times.

Nuclear maturation was assessed based on the extrusion of the first polar body, a marker of progression to metaphase II. After 19 h of IVM, oocytes from the control group (*n* = 631) and the treated groups [Lip‐blank (*n* = 648), 0.01 µg/mL Lip‐α‐pinene (*n* = 666), 1.0 µg/mL Lip‐α‐pinene (*n* = 655), and 100.0 µg/mL Lip‐α‐pinene (*n* = 625)] were denuded from cumulus cells by enzymatic and mechanical dissociation using TrypLE^TM^ Express Enzyme (Ferronato et al. 2023). The presence of the first polar body was evaluated under an inverted microscope. Following assessment, the oocytes returned to complete 22–24 h IVM period.

At the end of IVM, mature oocytes with an extruded polar body were washed in TCM‐199 medium supplemented with 10% FBS, 0.2 mM pyruvate, and 50 µg/mL gentamicin sulfate. Parthenogenetic activation was then performed chemically using a sequential protocol: first, the oocytes were incubated with 5 µM ionomycin for 5 min, followed by treatment with 2 mM 6‐dimethylaminopurine (6‐DMAP) for 3 h at 38.5°C in a 5% CO_2_ atmosphere (Sangalli et al. 2022). Following activation, the oocytes were cultured in 500 µL of synthetic oviductal fluid (SOF) medium supplemented with 2.5% FBS, 5 mg/mL bovine serum albumin (BSA), and 50 µg/mL gentamicin sulfate. Culture was conducted for 7 days at 38.5°C, in a controlled atmosphere of 5% CO_2_, 5% O_2_, and high humidity.

Cleavage rates were assessed on day 3 (48 h pos‐activation) using an inverted microscope (Nikon Eclipse, TS100, Tokyo, Japan). On day 7, the rated of initial blastocyst (iB), blastocyst (B), expanded blastocyst (eB), and hatched blastocyst (hB) formation were evaluated under the same microscope model.

### 2.6 Analysis of Reactive Oxygen Species (ROS) and Reduced Glutathione (GSH) Levels in In Vitro Matured Oocytes

The intracellular levels of ROS were quantified in denuded oocytes after 22 h of in vitro maturation using the fluorescent probe 2′,7′‐dichlorodihydrofluorescein diacetate (H_2_DCFDA; Invitrogen, Carlsbad, CA, USA). Oocytes from eight biological replicates per treatment group were washed in PBS containing 0.1% polyvinylpyrrolidone (PVP) and incubated with 5 µM H_2_DCFDA in PBS + 0.1% P for 30 min at 38.5°C in a 5% CO_2_ atmosphere. Following ROS staining, the same oocytes were incubated with 10 µM Cell Tracker^TM^ Blue CMF2HC (Molecular Probes, Thermo Fisher Scientific, USA) for an additional 30 min under identical conditions to assess intracellular GSH levels. After staining, oocytes were washed in PBS + 0.1% PVP, fixed in 4% paraformaldehyde for 15 min, and mounted on glass slides under coverslips for imaging.

Fluorescence analysis was performed using THUNDER Imaging System fluorescence microscope (Leica Microsystems, Wetzlar, Germany) equipped with a 40× objective. Excitation and emission filters were adjusted according to the specific fluorescence spectra of each dye. Images were acquired using identical microscope settings and sequential acquisition protocols for all samples. One image from the central plane of each oocyte was captured and analyzed using ImageJ software (NIH; http://rsb.info.nih.gov/ij/). Fluorescence intensity was measured and used as an indicator of intracellular ROS and GSH content (Del Collado et al. 2017).

### 2.7 Levels of mRNA in In Vitro Matured Oocytes

Analysis of mRNA levels was performed using pools of 20 oocytes per treatment from four biological replicates. To eliminate cumulus cells contamination, oocytes were denuded and had their zona pellucida removed by adding Tyrode's acid solution prior to RNA extraction. Total RNA was extracted using the Trizol® purification kit (Invitrogen, São Paulo, Brazil) with an on‐column DNase digestion step using PureLink^TM^ DNase (Thermo Fisher Scientific, USA), following the manufacturer's instructions. The concentration and purity of the RNA were assessed using a spectrophotometer (BioDrop, Cambridge, England) by measuring absorbance at 260 nm and checking the 260/280 ratio. For each sample, 1.6 μg of total RNA was used for first‐strand cDNA synthesis in a 30 µL reaction volume, using the Superscript III reverse transcriptase kit (Invitrogen, Waltham, Massachusetts, EUA) and random primers (Invitrogen), according to the manufacturer's instructions. The synthetized cDNA was stored at −20°C until used for quantitative PCR analysis.

The qPCR reactions were carried out using SYBR Green Master Mix (Applied Biosystems, Warrington, UK) on a StepOnePlus Real‐Time PCR System (Applied Biosystems, Foster City, CA, USA). The cycling conditions were: initial denaturation and enzyme activation at 95°C for 10 min, followed by 40 cycles of 95°C for 15 s, 58°C for 30 s, and 72°C for 30 s. A final extension step was performed at 72°C for 10 min. Primers were designed to amplify transcript from genes associated with oxidative stress superoxide dismutase (*SOD*), catalase (*CAT*), peroxiredoxin 6 (*PRDX6*), glutathione peroxidase 1 (*GPX1*), kelch‐like ECH‐associated protein 1 (*KEAP1*) and nuclear factor erythroid 2‐related factor 2 (*NRF2*) and endoplasmic reticulum stress markers (inositol requiring enzyme‐1 [*IRE*], kinase RNA‐like endoplasmic reticulum kinase [*PERK*] and transcription factor 6 [*ATF6*]). Glyceraldehyde‐3‐phosphate dehydrogenase (*GAPDH*) was used as the housekeeping gene (Table 1). All samples were run in duplicate, with a negative control lacking cDNA included in each run. The 2^‐ΔΔCt^ method was used to calculate relative mRNA expression levels (Livak and Schmittgen 2001).

**Table 1:** Primers used for amplification of messenger RNAs.

Gene	Primer sequence (5′ → 3′)	Sense (S) anti‐sense (As)	GenBank accession n^o^.
*GAPDH*	TGTTTGTGATGGGCGTGAACCAATG	S	GI: 402744670
	GCGCGTGGACAGTGGTCATAA	AS	
*PRDX6*	GCACCTCCTCTTACTTCCCGGA	S	GI: 59858298
	TGCGGCCGATGGTAGTAT	AS	
*GPX1*	AACGTAGCATCGCTCTGAGG	S	GI: 156602645
	GATGCCCAAACTGGTTGCAG	AS	
*SOD*	GTGAACAACCTCAACGTCGC	S	GI: 31341527
	GGGTTCTCCACCACCGTTAG	AS	
*CAT*	AAGTTCTGCATCGCCACTCA	S	GI: 402693375
	GGGGCCCTACTGTCAGACTA	AS	
*NRF2*	GACCCAGTCCAACCTTTGTC	S	GI: 0304941
	GACCCGGACTTACAGGTACT	AS	
*KEAP1*	TCACCAGGGAAGGATCTACG	S	Bt03817661_m1
	AGCGGCTCAACAGGTACAGT	AS	
*IRE1*	ATGAGGAGACCGGCATGGTG	S	XM_010824247.1
	AAACATGCCCCGGTACACGA	AS	
*PERK*	CCCGTTCGGCACTCAAATGG	S	NM_001098086.1
	TGCACCATGGCATACTCACGA	AS	
*ATF6*	AGAGGAGCAAGACACATCGG	S	XM_003585816.2
	CAGTTAGCACCATCAGGGCT	AS	

### 2.8 Ultrastructural Analysis of In Vitro Matured Cumulus‐Oocyte Complexes

Ultrastructural evaluation of COCs was performed as previously described by Silva et al. (2024). After IVM, COCs (*n* = 5–7 per treatment) were fixed in Karnovsky solution (2% paraformaldehyde and 2% glutaraldehyde in 0.1 M sodium cacodylate buffer, pH 7.2). Post‐fixation was conducted in 1% osmium tetroxide, 0.8% potassium ferricyanide, and 5 mM calcium chloride in 0.1 M sodium cacodylate buffer for 1 h. Following buffer washes, samples were stained with 5% uranyl acetate, dehydrated in acetone, and embedded in epoxy resin (Epoxy‐Embedding Kit, Fluka Chemika‐ BioChemika). Semi‐thin sections (2 μm) were cut, stained with toluidine blue, and observed under light microscopy at 400× magnification. Ultra‐thin sections (70 nm) were subsequently obtained, stained with uranyl acetate and lead citrate, and analyzed under a transmission electron microscope (Fei Tecnai Spirit, North American Sales, USA).

### 2.9 Lipid Content in Oocytes and Embryos and Total Cell Number in Blastocysts

Lipid content in matured oocytes and 7‐day cultured embryos was evaluated using the neutral lipid‐specific dye BODIPY 493/503 (Molecular Probes, USA). Denuded oocytes and embryos (*n* = 45–50 per group) were fixed in 4% PFA for 15 min, permeabilized with 0.1% saponin for 30 min, and washed with PBS containing 0.1% PVP. Samples were stained with 20 µg/mL BODIPY for 60 min at room temperature, protected from light, then washed and mounted on glass slides using ProLong Gold mounting medium.

Samples were analyzed using a confocal laser scanning microscope (LSM 710, Carl Zeiss, Oberkochen, Germany) with a 63x oil immersion objective. An argon laser was used for excitation at 388 nm, and emission was collected between 500 and 537 nm. One image per oocyte or embryo was taken at the section with the largest diameter. ImageJ software (NIH; http://rsb.info.nih.gov/ij/) was used to quantify the ratio of lipid droplet area to total cell area, as previously described by Del Collado et al. (2015). Images were converted to 8‐bit, and lipid droplet area was calculated using the “nucleus counter” plugin. Lipid droplet sizes were categorized as < 2.0 µm, 2.0–6.0 µm, and > 6.0 µm (Abe et al. 2002). For blastocysts, lipid content was normalized to total embryo area (Del Collado et al. 2015). The total number of cells per blastocyst was determined using Hoechst 33342 staining.

### 2.10 Statistical Analysis

Statistical analyses were conducted using GraphPad Prism version 9.0. Data normality was assessed with the Shapiro‐Wilk test. Student's t‐test was used to compare the percentage of viable CCs among treatments. The Chi‐square test was used to evaluate differences in oocyte maturation rates, embryo cleavage, and blastocyst formation across treatments. The levels of mRNA, lipid droplet size, and total cell number in blastocysts were analyzed using two‐way ANOVA, followed by Tukey's post‐hoc test. Lipid content in oocytes and embryos, as well as ROS and GSH levels, were compared using the non‐parametric Kruskal‐Wallis test. Spearman's correlation was used to evaluate the association between ROS and GSH levels at the individual oocyte level. Results were considered statistically significant when *p* < 0.05. Data are presented as mean ± standard error of the mean.

## 3 Results

### 3.1 Characterization of Liposomes

The hydrodynamic particle size, polydispersity index (PDI), and zeta potential (ZP) are crucial parameters that influence the stability and colloidal behavior of liposome nanocarriers. In this context, Lip‐blank formulation showed a mean diameter of 130.82 ± 1.32 nm, PDI of 0.29 ± 0.02 and ZP of −13.78 ± 1.08 mV. In contrast, Lip‐α‐pinene exhibited a smaller mean diameter of 75.86 ± 0.95 nm, PDI of 0.26 ± 0.00, a higher negative charge (ZP of −31.55 ± 2.23 mV), and encapsulation efficiency of 85.00 ± 2.51%.

**Figure 1:**
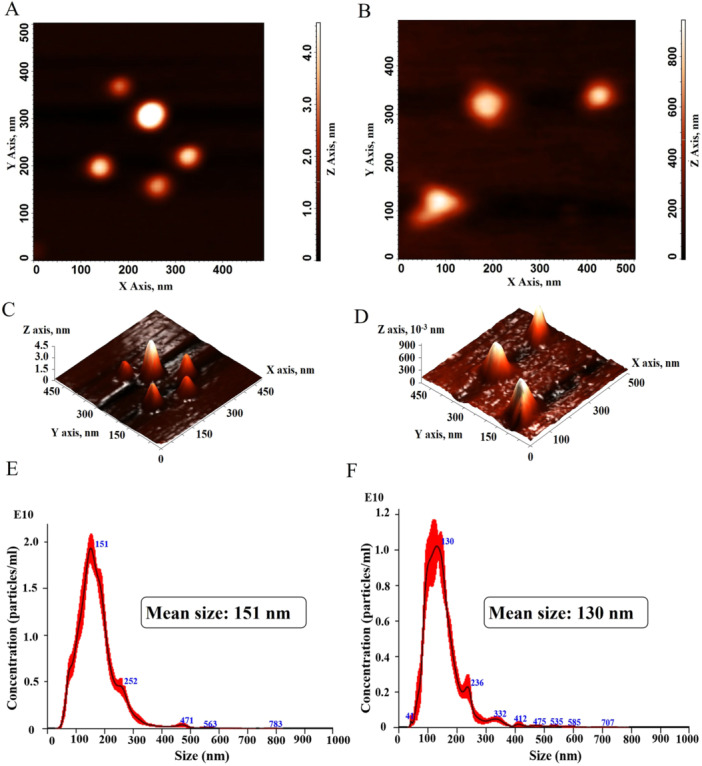
Atomic Force Microscopy and Nanotracking analysis. Two‐dimensional (A, B) and three‐dimensional (C, D) from atomic force microscopy (AFM) micrographs of Lip‐blank and Lip‐α‐pinene, respectively. Concentration/Size graphs for NTA of particles in Lip‐blank (E) and Lip‐α‐pinene (F).

The AFM images revealed the presence of some larger particles in the Lip‐blank formulation, likely due to aggregation of smaller vesicles. However, this did not significantly affect the particle size distribution, with a measured mean size of 133 nm (Figure 1A and C). In contrast, Lip‐α‐pinene showed uniform particle distribution with peaks of similar height and a mean size of 77 nm (Figure 1B and D). The NTA confirmed the particle concentration and size: Lip‐blank had 2.38 ×1012 particles/mL with a mean size of 150 nm, while Lip‐α‐pinene had 1.15 ×1012 particles/mL and mean size 129.7 nm (Figure 1E and F).

**Figure 2:**
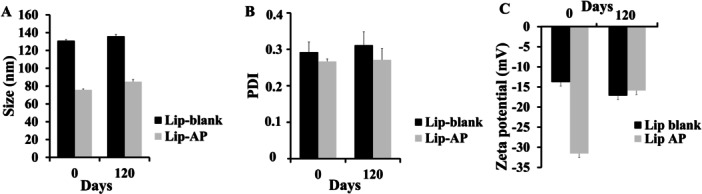
Physical parameters such as size, PDI and zeta potential. The average size (in diameter) (A), PDI indices (B), zeta potential (C) of Lip‐blank and Lip‐α‐pinene (AP) immediately after the fabrication or 120 days later (stored in 4°C) (mean ± SD, n = 3).

Regarding storage stability, both liposomal formulations remained stable up to 120 days at 25°C. After this period, Lip‐blank had a mean particle size of 135.86 ± 2.28 nm, PDI of 0.31 ± 0.03, and ZP of −17.14 ± 0.17 mV. Lip‐α‐pinene maintained a particle size of 84.98 ± 2.30 nm, PDI of 0.27 ± 0.03, and ZP of −15.89 ± 0.07 mV, with no significant variation over time as shown in Figure 2. The addition of α‐pinene seemed to reduce the particle size, improved homogeneity, and increased negative charge, all of which contributed to the enhanced physical stability of the formulation.

### 3.2 Effect of Liposome Supplementation on Bovine Cumulus Cells Viability

**Figure 3:**
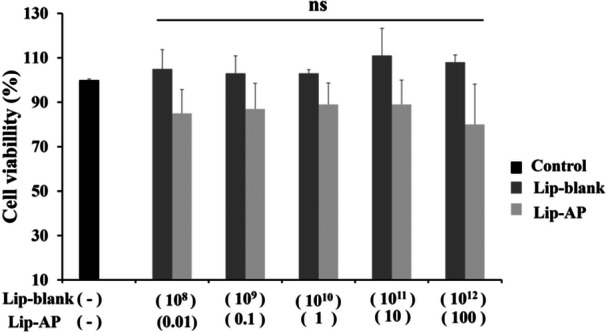
Cell toxicity analysis. Effect of 24 h exposure to different concentrations of Lip‐blank and Lip‐α‐pinene (AP) at 10^8^ to 10^12^ particles/mL on the viability of cumulus cell line assessed by MTT assay. For Lip‐α‐pinene, this meant α‐pinene concentrations ranging from 0.01 to 100.0 µg/mL.

Neither Lip‐blank or Lip‐α‐pinene exhibited cytotoxic effects on cumulus cells. After 24 h exposure to increasing concentrations of Lip‐blank and Lip‐α‐pinene (10^8^ to 10^12^ nanoparticles/mL), no significant difference was found between the groups and concentrations (*p* > 0.05, Figure 3).

### 3.3 Effect of Lip‐α‐Pinene Supplementation on Nuclear Maturation of Bovine Oocytes

The nuclear maturation rate of oocytes significantly increased when matured with 1.0 µg/mL Lip‐α‐pinene (78.5 ± 0.90%), compared to those treated with Lip‐blank (71.0 ± 0.84%) or 100.0 µg/mL Lip‐α‐pinene (72.0 ± 1.04%) (*p *< 0.05). However, no significant differences were observed between the control group (75.0 ± 0.89%) and the 0.01 µg/mL Lip‐α‐pinene group (76.0 ± 0.75%) (*p *> 0.05, Table 2).

**Table 2:** Rate of matured bovine oocytes after 22 h of in vitro maturation in the presence of different supplements (Lip‐blank and Lip‐α‐pinene (AP) at 0.01 µg/mL, 1.0 µg/mL, and 100.0 µg/mL).

Groups	No. oocytes cultured	Oocytes with first polar body	Extrusion proportion (% + SEM)
Control	631	471	75 ± 0.89^a,b,c^
Lip‐blank	648	459	71 ± 0.84^a^
Lip‐AP 0.01	666	504	76 ± 0.75^b,c^
Lip‐AP 1.0	655	512	78.5 ± 0.90^b^
Lip‐AP 100.0	625	457	72 ± 1.04^a,c^

*Note:* a,b, c values with different superscripts within a column are significantly different (*p* < 0.05). Results from 14 replicates. Means presented with mean standard error.

### 3.4 Effects of Lip‐α‐Pinene on ROS and GSH Levels in Matured Oocytes

**Figure 4:**
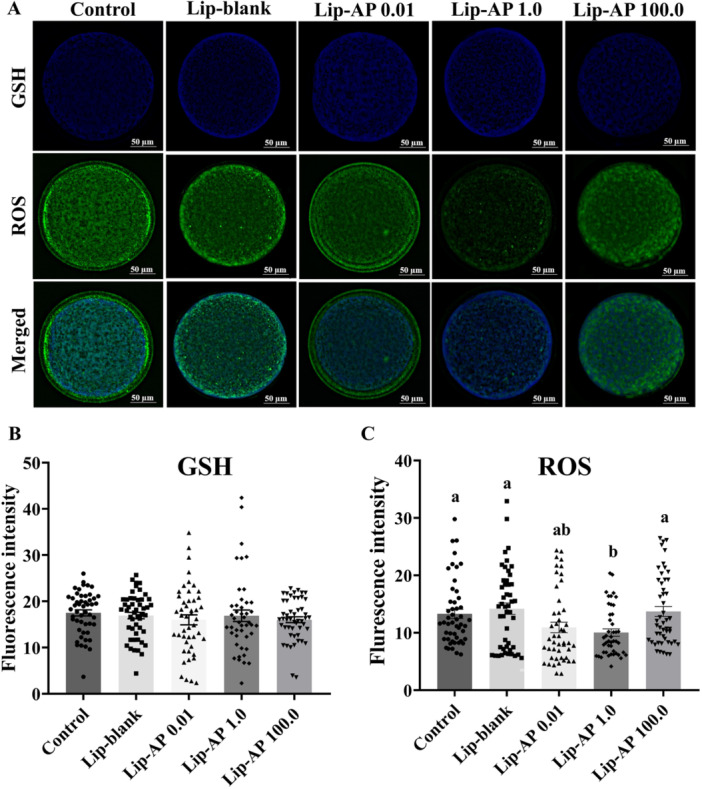
GSH and ROS analysis. Representative image of bovine oocytes stained with Cell Tracker Blue (GSH) and H_2_DCFDA (ROS) in green after 22 h IVM (A). Quantification of GSH fluorescence intensity (B) and ROS (C) in oocytes matured in vitro in presence of Lip‐blank and Lip‐α‐pinene (AP) at 0.01 µg/mL, 1.0 µg/mL, and 100.0 µg/mL. 45‐50 oocytes/treatment were evaluated. Different letters above the bars the graph indicate significant differences (*p* < 0.05). Bars depict the means and error bars depict S.E.M.

The addition of Lip‐blank or 0.01, 1.0, or 100.0 µg/mL Lip‐α‐pinene during maturation did not affect intracellular GSH levels (*p* > 0.05, Figure 4A and B). However, oocytes treated with 1.0 µg/mL Lip‐α‐pinene exhibited significantly reduced intracellular ROS levels compared to the control, Lip‐blank, and 100.0 µg/mL Lip‐α‐pinene (*p *< 0.05, Figure 4A and C). The correlation analysis between ROS and GSH levels in each oocyte showed no significant association across groups, except for 0.01 µg/mL Lip‐α‐pinene, which displayed a significant positive correlation (*p *< 0.05, Supporting Figure S1).

### 3.5 Effects of Lip‐α‐Pinene on Oocyte Lipid Droplet Content

**Figure 5:**
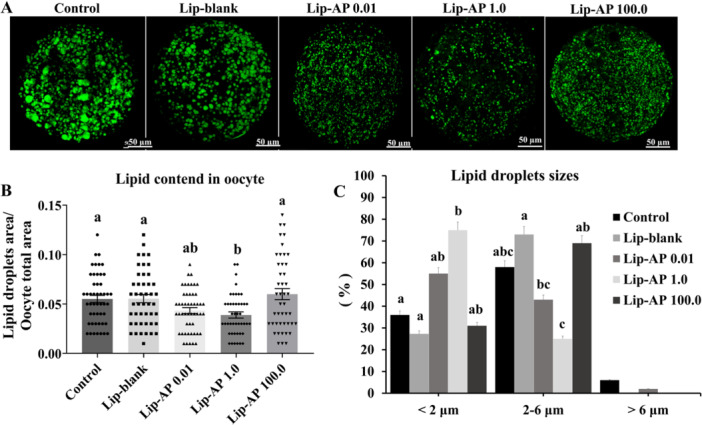
Oocyte lipid droplets analysis. Representative image of bovine oocytes stained with Bodipy 493/503 after 22 h IVM (A). Quantification of cytoplasmatic lipid droplet content over the total oocyte area (B). Lipid‐droplet size classification of oocyte after IVM in presence of Lip‐blank and Lip‐α‐pinene (AP) at 0.01 µg/mL, 1.0 µg/mL, and 100.0 µg/mL (C). 45–50 oocytes/treatment were evaluated. Different letters above the bars the graph indicate significant differences (*p* < 0.05). Bars depict the means and error bars depict S.E.M.

Oocytes matured in the presence of 1.0 µg/mL Lip‐α‐pinene showed lower lipid content compared to those cultured in control medium, Lip‐blank, or 100.0 µg/mL Lip‐α‐pinene (*p *< 0.05), with levels similar to the 0.01 µg/mL Lip‐α‐pinene (Figure 5A and B). Additionally, 1.0 µg/mL Lip‐α‐pinene increased the proportion of small lipid droplets (< 2 µm) and reduced the proportion of large droplets (2–6 µm), compared to Lip‐blank and 100.0 µg/mL Lip‐α‐pinene groups (*p* < 0.05, Figure 5C).

### 3.6 Effects of Lip‐α‐Pinene on Embryo Development and Lipid Content

**Figure 6:**
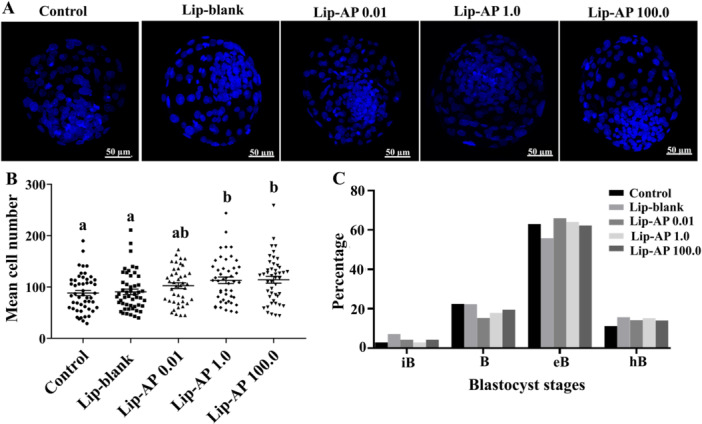
Blastocyst cell counts. Representative images of blastocysts stained with Hoechst 33342 (nuclei marker) in parthenogenic embryos derived from bovine oocytes matured in vitro in presence of Lip‐blank and Lip‐α‐pinene (AP) at 0.01 µg/mL, 1.0 µg/mL, and 100.0 µg/mL (A). Total cell number in blastocyst IVC (*n* = 43–50 blastocyst/treatment were evaluated). Different letters show statistically significant difference between treatments (*p* < 0.05). Date are the means ± SEM. Percentage of blastocyst stages on day 7 post‐PA: initial blastocyst (iB), blastocyst (B), Expanded blastocyst (eB), and hatching or hatched blastocyst (hB) (C).

**Figure 7:**
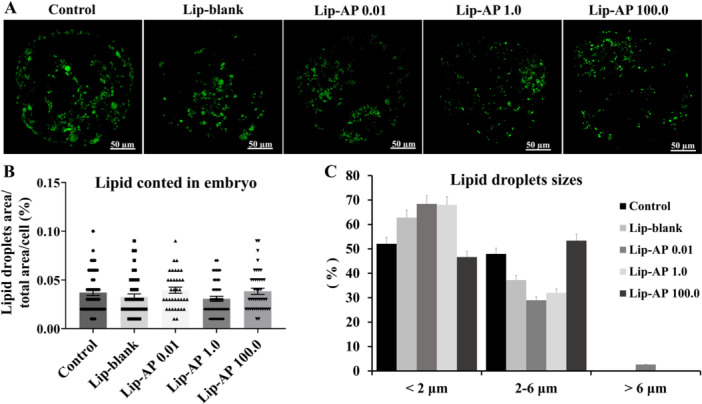
Embryo lipid droplets analysis. Representative image of parthenogenetic bovine blastocysts stained with Bodipy 493/503 after 7 days ICV (A) Quantification of intracytoplasmic lipid content over the total blastocyst area (B). Lipid‐droplet size classification of blastocysts derived from oocytes matured in vitro in the presence of Lip‐blank and Lip‐α‐pinene (AP) at 0.01 µg/mL, 1.0 µg/mL, and 100.0 µg/mL (C). A total of 45–50 blastocyst/treatment were evaluated. Different letters above the bars the graph indicate significant differences (*p* < 0.05). Bars depict the means and error bars depict S.E.M.

Supplementation of culture medium with 1.0 µg/mL Lip‐α‐pinene during oocyte maturation significantly increased cleavage rates compared to control medium alone or supplemented with 0.01 µg/mL Lip‐α‐pinene (*p* < 0.05, Table 3). However, no significant differences were observed in blastocysts rates among treatments (*p *> 0.05). Blastocyst from oocytes matured with 1.0 and 100.0 µg/mL Lip‐α‐pinene had greater number of cells than those matured in control medium alone or supplemented with Lip‐blank (*p *< 0.05, Figure 6A and B). The distribution of blastocyst developmental stages (iB, B, eB, and hB) did not differ between treatments (*p *> 0.05, Figure 6C). Lipid content and lipid‐droplet size in blastocysts were also not affected by Lip‐α‐pinene (Figure 7A and B).

**Table 3:** Cleavage and blastocyst rates after of IVM of bovine oocytes in medium supplemented with Lip‐blank and Lip‐α‐pinene (AP) at 0.01 µg/mL, 1.0 µg/mL, and 100.0 µg/mL.

Groups	ICV (*n*)	Cleavage *n* (% ± SEM)	Blastocyst *n* (% ± SEM)
Control	201	121 (59.08 ± 1.46) ^ac^	79 (40.16 ± 1.65)
Lip‐blank	196	135 (67.91 ± 1.21) ^ab^	77 (38.16 ± 2.02)
Lip‐AP 0.01	201	105 (61.70 ± 1.66) ^c^	67 (39.40 ± 1.09)
Lip‐AP 1.0	209	149 (71.16 ± 0.95) ^b^	92 (44.00 ± 1.61)
Lip‐AP 100.0	191	139 (72.16 ± 1.95) ^b^	75 (41.66 ± 1.22)

*Note:* a,b,c values with different superscripts within a column are significantly different (*p* < 0.05). Results from 6 replicates. Means presented with mean standard error.

### 3.7 Effects of Lip‐α‐Pinene on Expression of mRNA for Proteins Related to Oxidative and Endoplasmic Reticulum Stress

**Figure 8:**
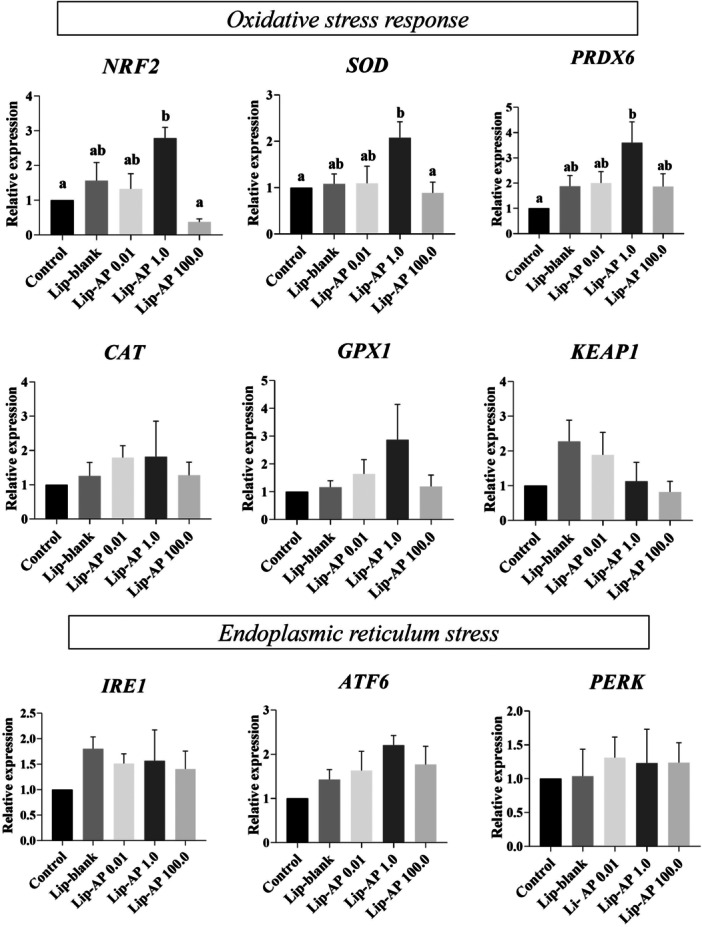
Relative mRNA abundance of oxidative stress and endoplasmic reticulum stress‐related genes in oocyte after IVM. Oocytes were matured in vitro in presence of Lip‐blank and Lip‐α‐pinene (AP) at 0.01 µg/mL, 1.0 µg/mL, and 100.0 µg/mL and the levels of *CAT*, *SOD*, *GPX1*, *PRDX6*, *NRF2, KEAP1*, *IRE1*, *ATF6* and *PERK* were evaluated by real time PCR. Different letter above the bars for the same genes indicate significant differences (*p* < 0.05). Bars depict the means and error bars depict S.E.M.

Oocytes matured with 1.0 µg/mL Lip‐α‐pinene had significantly increased mRNA levels for *NRF2* when compared to those matured in control medium alone or supplemented with 100.0 µg/mL Lip‐α‐pinene (*p* < 0.05). Similarly, *SOD* expression increased in oocytes matured with 1.0 µg/mL Lip‐α‐pinene when compared to those matured in control or 100.0 µg/mL Lip‐α‐pinene (*p* < 0.05), but did not differ from other treatments (*p* > 0.05). The *PRDX6* transcript levels were higher in oocytes cultured with 1.0 µg/mL Lip‐α‐pinene than those from control group (*p* < 0.05), but showed no difference with other treatments (*p* > 0.05). No significant changes were observed in the expression of *CAT*, *GPX1*, *KEAP1, IRE1*, *ATF6*, and *PERK* genes among treatments (*p* > 0.05, Figure 8).

### 3.8 Effects of Lip‐α‐Pinene on Ultrastructure of COCs

**Figure 9:**
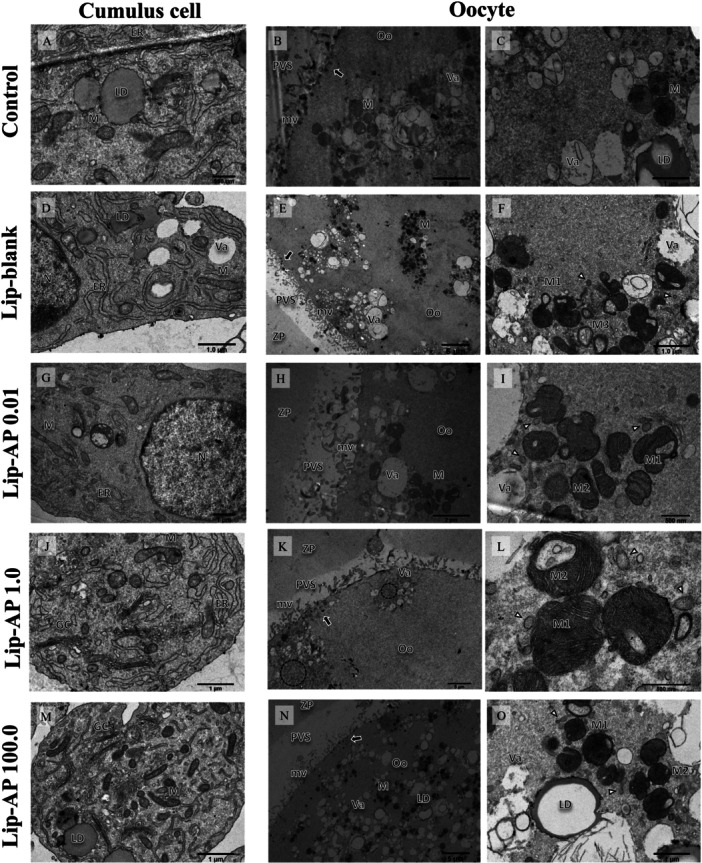
Transmitted electron microscopy analysis. Electron micrograph of oocyte and cumulus cells matured in control (A–C), Lip‐blank (D–F), 0.01 µg/mL Lip‐α‐pinene (G–I), 1.0 µg/mL Lip‐α‐pinene (J–L) and 100.0 µg/mL Lip‐α‐pinene (M–O). Cumulus cells in all groups exhibited normally distributed mitochondria (M), endoplasmic reticulum (ER), Golgi complex (GC), and lipid droplets (LD). Oocytes from all groups exhibited microvilli (mv) projecting into the perivitelline space (PVS), an intact zona pellucida (ZP), and mitochondria arranged in clusters showing transversal cristae (M2), eccentric cristae (M3), and, in the Lip‐blank group, some annular‐shaped mitochondria (M3). A high density of vacuoles (Va) of varying sizes was observed in the oolemma (Oo) of oocytes from all groups, except for those treated with 1.0 µg/mL Lip‐α‐pinene, which exhibited only a few vacuoles. Additionally, oocytes from this group showed clusters of endoplasmic reticulum vesicles (black circle), and mitochondria exhibited a hooded morphology. AP: α‐pinene. N: nucleus; White arrowheads: cisternae of smooth and rough endoplasmic reticulum; Black arrows: Cortical granules.

The cumulus cells and oocyte from all treatments exhibited normal and well‐preserved ultrastructural features. The cumulus cells contained elongated mitochondria, well‐developed endoplasmic reticulum and Golgi complex, lipid droplets and few vacuoles. The oocytes had an intact zona pellucida and a continuous layer of long, thin microvilli projecting from the oolemma surface into the perivitelline space. Mitochondria had a hooded shape, with thin cristae arranged transversely or eccentrically, and were often observed in clusters or in proximity to endoplasmic reticulum vesicles. These features were evident in all treatments except the control group. Enucleated mitochondria were observed only in oocytes treated with Lip‐blank. Isolated cortical granules located at the periphery of the oocyte were present in all treatments, except in the group treated with 0.01 µg/mL Lip‐α‐pinene. A high density of electron‐pale vacuoles was observed in all treatments, except in the oocytes treated with 1.0 µg/mL Lip‐α‐pinene, which exhibited a small number of vacuoles. Additionally, the oocytes from this group exhibited clusters of endoplasmic reticulum vesicles in the peripheral region of the oolemma (Figure 9).

## 4 Discussion

This study is the first to examine α‐pinene encapsulation in liposomes as a strategy to improve bovine oocyte in vitro maturation through antioxidant mechanisms. Liposomes, widely recognized as effective molecular delivery systems, are particularly suitable for transporting lipophilic antioxidants, such as α‐pinene. They help to reduce cytotoxicity, enhance cellular uptake, and minimize oxidative stress‐associated damage (Jasim et al. 2024).

Our results revealed that Lip‐α‐pinene exhibited high encapsulation efficiency (85.00 ± 2.51%) and remained stable over 4 months. The observed negative zeta potential may have contributed to the electrostatic repulsion between liposomes, thereby stabilizing the colloidal dispersion and preventing particle aggregation (Jasim et al. 2024). Additionally, the Lip‐α‐pinene particle size ranged from 75.86 to 84.98 nm, which falls within the nanoscale range (50–200 nm) commonly associated with increased dispersion stability and efficient interaction with biological systems (Nsairat et al. 2022). Given the anionic nature of Lip‐α‐pinene, which can create electrostatic repulsion with the negatively charged cell membrane (Tseu and Kamaruzaman 2023), potentially limiting its direct internalization, we believe that Lip‐α‐pinene may have acted as a controlled release system, gradually delivering α‐pinene into the maturation medium, increasing its bioavailability and preventing potential cytotoxic effects from high concentrations. Previous studies have already demonstrated α‐pinene cytotoxicity at high concentrations in cell culture (Porres‐Martínez et al. 2015; Karthikeyan et al. 2018; Xanthis et al. 2021). However, we can not exclude the possibility that Lip‐α‐pinene can be internalized in cells by endocytotic mechanisms, such as clathrin or caveolae mediated endocytosis (Mateos‐Maroto et al. 2023). Clathrin dependent endocytosis has already been reported in both cumulus cells and oocytes (Hölzenspies et al. 2010). In addition, the phospholipids used in the composition of anionic liposomes are phospholipids found naturally in cell membranes. This composition allows anionic liposomes, when used as a bioactive delivery system, to fuse directly with the biological membrane, so that they do not form an endosome within the membrane and release their cargo directly into the cytosol (Tseu and Kamaruzaman 2023), supporting the possibility that Lip‐α‐pinene may also have been internalized via endocytosis or by fusion direct with the biological membrane, which could have contributed to the intracellular delivery of α‐pinene, potentially enhancing its antioxidant activity during IVM.

Consistent with this hypothesis, our findings indicated that both Lip‐blank (without α‐pinene) and Lip‐α‐pinene did not exert cytotoxic effects on bovine cumulus cells. The safety and efficacy of natural antioxidants encapsulated in liposomes have also been confirmed by other studies. Caddeo et al. (2021) demonstrated that liposomal resveratrol protected 3T3‐L1 mouse fibroblast cells from cytotoxicity, even at high doses, where resveratrol may act as either an antioxidant or a pro‐oxidant. Similarly, De Luca et al. (2022) reported that *Myrtus communis L. (myrtle)* extract encapsulated in liposomes showed no cytotoxicity or increase in ROS levels after 24 h of exposure in the same cell line. Chen et al. (2024) also reported that liposome‐encapsulated quercetin increased the viability of PC12 and bEnd.3 cells due to its controlled‐release properties, which resulted in lower levels of free quercetin and consequently reduced cytotoxicity.

The oocyte nuclear maturation rate, assessed by the extrusion of the first polar body, was not significantly affected by the addition of Lip‐α‐pinene to the IVM medium. This finding aligns with previous studies demonstrating that antioxidant supplementation during IVM does not directly influence nuclear maturation in bovine oocytes, as previously demonstrated with carvacrol (Morais et al. 2023), eugenol (Silva et al. 2022), and anethole (Sá et al. 2020). Nevertheless, natural antioxidants have been suggested to enhance oocyte cytoplasmic maturation and subsequent embryonic development by promoting the storage of mRNAs, including those encoding antioxidant enzymes (Rodrigues‐Cunha et al. 2016), a mechanism that appears to be supported by our results.

We observed that supplementation of culture medium with 1.0 µg/mL Lip‐α‐pinene during IVM significantly reduced intracellular ROS levels in bovine oocytes. ROS is a central mediator of intrinsic oxidative stress pathways (Kimura et al. 2010). Oxidative stress, defined as a shift from a physiological to a pro‐oxidant redox state, can lead to cellular damage when antioxidant defense mechanisms fail (Perkins et al. 2020). Previous studies have shown that α‐pinene reduces ROS generation induced by oxidative insults, such as UVA exposure in human keratinocytes (Karthikeyan et al. 2018) and H_2_O_2_ in PC12 cells (Porres‐Martínez et al. 2016). The encapsulation of α‐pinene in liposomes provides a controlled release system in the maturation medium, and due to its high lipophilicity, α‐pinene likely interacts efficiently with cell membranes (Elmann et al. 2009), enhancing its ability to scavenge peroxyl radicals involved in lipid peroxidation (Porres‐Martínez et al. 2015). In our study, we observed no significant association between ROS and GSH levels across groups, except for 0.01 µg/mL Lip‐α‐pinene, which showed a positive correlation, indicating a specific antioxidant response. This suggests that low concentrations may trigger compensatory GSH responses, while 1.0 µg/mL Lip‐α‐pinene selectively suppresses ROS without altering GSH homeostasis. These findings suggest that Lip‐α‐pinene at 1.0 µg/mL may optimize the antioxidant effects of α‐pinene, thereby reducing ROS levels and enhancing both oocyte competence and parthenogenetic embryo quality. However, a segment of the analysis according to nuclear stage (e.g., MII and non‐MII) should be incorporated in future studies when assessing ROS and GSH levels to elucidate whether α‑pinene–loaded liposomes exert a more pronounced effect specifically fully matured oocyte. Additionally, GSH and ROS levels were not measured in cumulus cells, a limitation given their redox regulation role during oocyte IVM. Ban et al. (2025) showed cumulus cells co‐cultured with oocytes significantly reduce ROS levels, suggesting Lip‐α‐pinene exerted indirect antioxidant effects through cumulus metabolism.

Although high GSH levels in bovine oocytes are known to mitigate ROS production (Sonjaya et al. 2025), our study showed that GSH levels in the Lip‐α‐pinene group were similar to those in the control. This suggests that the observed antioxidant effect may be mediated through enzymatic rather than non‐enzymatic mechanisms. Supporting this, the analysis of oxidative stress‐related transcripts revealed significant changes in the relative abundance of *NRF2*, *SOD*, and *PRDX6* mRNAs following treatment with 1.0 µg/mL Lip‐α‐pinene in vitro matured bovine oocytes. These findings are consistent with prior studies indicating that α‐pinene increases *NRF2* and antioxidant enzymes levels, highlighting its role in regulating oxidative stress through indirect mechanisms (Porres‐Martínez et al. 2016; Xanthis et al. 2021). *NRF2* is a key transcription factor in the cellular adaptive response to oxidative stress, playing a protective role in reproductive cells by enhancing antioxidant defense genes (Khan et al. 2024). *SOD* catalyzes the conversion of superoxide into hydrogen peroxide (Xu et al. 2022), while *PRDX6* reduces phospholipid hydroperoxides and exhibits both phospholipase A2 and lysophosphatidylcholine acyltransferase activities (Fisher et al. 2018). *PRDX6* has also been implicated in oxidative defense and intracellular signaling in bovine oocytes through its alkyl and hydrogen peroxide reductase functions (Leyens et al. 2004). Thus, we suggest that the higher abundance of these transcripts may indicate an enhanced ability of oocytes from the group treated with 1.0 µg/mL Lip‐α‐pinene to preserve and stabilize mRNAs during IVM, making them more available for the synthesis of antioxidant enzymes. This mechanism may have contributed to the reduction of ROS levels and, ultimately, to the improvement of embryo quality after parthenogenetic activation.

Oocyte maturation with 1.0 µg/mL Lip‐α‐pinene led to a reduction in cytoplasmic lipid accumulation and altered lipid droplet size and distribution, resulting in a more homogeneous and centrally located lipid pattern. Lipids are important signaling molecules involved in regulatory mechanisms during oocyte maturation and competence acquisition (Dunning et al. 2014). However, excessive lipid accumulation, often a byproduct of in vitro culture, can disrupt cellular homeostasis and increase susceptibility to oxidative stress (Rizos et al. 2003; Ordoñez‐Leon et al. 2014). The use of FBS in IVM media is known to induce lipid accumulation and metabolic alterations in COCs (Del Collado et al. 2015). On the other hand, antioxidants such as vitamin E or cysteamine, especially when combined with eicosapentaenoic acid, have been shown to reduce lipid droplet accumulation in bovine oocytes (Nikoloff et al. 2020). This supports the idea that antioxidant presence during IVM may influence lipid metabolism, thereby decreasing both lipid accumulation and ROS levels. In porcine oocytes, Somfai et al. (2011) observed that reduced lipid droplets were associated with increased mitochondrial activity and decreased ROS, further supporting this link. Thus, Lip‐α‐pinene at 1.0 µg/mL may have contributed to a more stable intracellular environment, possibly promoting lipid utilization as an energy source during oocyte maturation. However, a lipid droplet analysis according to nuclear stage (MII and non‐MII) should be explored in future studies when assessing α‐pinene liposome effects to help understand whether this lipid modulation exerts a more pronounced effect specifically in fully matured oocytes. Notably, the lipid reduction observed in oocytes did not persist into the blastocyst stage, as no differences were observed in embryonic lipid content among treatments. This transient effect can align the distinct culture conditions, where IVM was performed under 21% O₂ with 10% FBS (lipid‐promoting), while embryonic IVC occurred under 5% O₂ with 2.5% FBS (lipid‐minimizing). This suggests that Lip‐α‐pinene specifically modulated lipid metabolism during IVM without long‐term embryonic effects.

Regarding the ultrastructural characteristics of the COCs matured in vitro in the presence of Lip‐α‐pinene, both cumulus cells and oocytes were well preserved, along with key important organelles such as mitochondria, endoplasmic reticulum and Golgi complex, as well as an intact zona pellucida and numerous microvilli, regardless of the concentration used, indicating maintenance of cell integrity during the IVM process. However, COCs matured in 1.0 µg/mL Lip‐α‐pinene exhibited a lower density of cytoplasmic vacuoles, suggesting a potential protective effect on oocytes cytoplasmic quality. Increased vacuolation in gametes is strongly associated with cellular stress conditions with failure in in vitro embryo production (Van Blerkom 1990) and is recognized as a sign of degeneration (Zamboni et al. 1972). Moreover, the presence of hooded mitochondria a morphological change more prevalent during the later stages of oocyte growth in cows (Lodde et al. 2008), and may be related to specific cellular functions, such as increased oxidative capacity (Suen et al. 2008). These findings may reflect a functional adaptation aimed at maintaining redox homeostasis, since 1.0 µg/mL of Lip‐α‐pinene was able to reduce intracellular ROS levels.

Regarding embryonic development, we observed that both 1.0 µg/mL and 100.0 µg/mL Lip‐α‐pinene influenced the cleavage rates and total cell number in blastocysts. However, Lip‐α‐pinene supplementation during IVM did not significantly affect the overall blastocyst formation rate. The total number of cells per blastocyst is a key indicator of embryo quality and is typically associated with reduced apoptosis and enhanced cytoplasmic maturation during IVM (Khan et al. 2018). When delivered via nanocarriers, antioxidants can reduce cytotoxicity, enhance bioavailability, and improve developmental outcomes (Remião et al. 2016). Therefore, our findings suggest that Lip‐α‐pinene supplementation during IVM may have positively impacted early embryonic development, enhancing embryo quality without necessarily promoting progression to the blastocyst stage. Future studies should explore the effects of continued Lip‐α‐pinene supplementation during subsequent embryo culture to fully elucidate its potential benefits throughout preimplantation development.

In conclusion, liposome‐encapsulated α‐pinene (1.0 μg/mL) enhances oocyte maturation by preserving the ultrastructure of oocyte and cumulus cells and reducing intracellular ROS levels and lipid accumulation. These effects appear to be mediated through the activation of the antioxidant defense system, including upregulation of *NRF2*, *SOD*, and *PRDX6*, contributing to improved oocyte competence and better‐quality parthenogenetic embryos. While Lip‐α‐pinene supplementation positively influenced early embryo development, its effect on blastocyst formation was limited. Overall, Lip‐α‐pinene appears to be a promising strategy to improve oocyte quality during in vitro maturation.

## Author Contributions

**Venância A.N. Azevedo:** conceptualization, methodology, investigation, formal analysis, data curation, validation, visualization, writing – original draft, writing – review and editing. **Maria A. Almeida:** investigation, visualization, writing – review and editing. **Matheus A. Chaves:** methodology, investigation, data curation, visualization, writing – original draft, writing – review and editing. **Luca A. Souza:** investigation, visualization, writing – review and editing. **Luiziana C.C.F. Crisóstomo:** methodology, investigation, data curation, visualization, writing – original draft, writing – review and editing. **Mariana A.M. Donato:** investigation, visualization, writing – review and editing. **Christina A. Peixoto:** investigation, visualization, writing – review and editing. **Josimar O. Eloy:** resources, methodology, data curation, validation, visualization, writing – original draft, writing – review and editing. **Flávio V. Meirelles:** resources, methodology, writing – review and editing. **Felipe Perecin:** resources, methodology, writing – review and editing. **Juliano C. Silveira:** resources, methodology, formal analysis, data curation, validation, visualization, writing – original draft, writing – review and editing. **José R.V. Silva:** conceptualization, methodology, formal analysis, validation, supervision, resources, funding acquisition, writing – original draft, writing – review and editing.

## Conflicts of Interest

The authors declare no conflicts of interest.

## Supporting information

Figure_1_Supplnfo. Correlation between ROS and GSH levels in bovine oocytes matured in vitro in the control (n = 50), Lip‐blank (n = 48) and Lip‐α‐pinene (AP) at 0.01 μg/mL (n = 47), 1.0 μg/mL (n = 45), and 100.0 μg/mL (n = 50).

## Data Availability

The authors have nothing to report.
