# Supplementary material for: Effects of Liposome‐Encapsulated α‐Pinene on In Vitro Oocyte Maturation and Embryo Development in Bovine Species
Source: Mol Reprod Dev. 2026 Feb 19;93(2):e70094. doi: 10.1002/mrd.70094 (PMC12917933; doi:10.1002/mrd.70094)
Supplement: Supplementary file 1 — Figure_1_Supplnfo. Correlation between ROS and GSH levels in bovine oocytes matured in vitro in the control (n = 50), Lip‐blank (n = 48) and Lip‐α‐pinene (AP) at 0.01 μg/mL (n = 47), 1.0 μg/mL (n = 45), and 100.0 μg/mL (n = 50). [file MRD-93-e70094-s001.pdf]

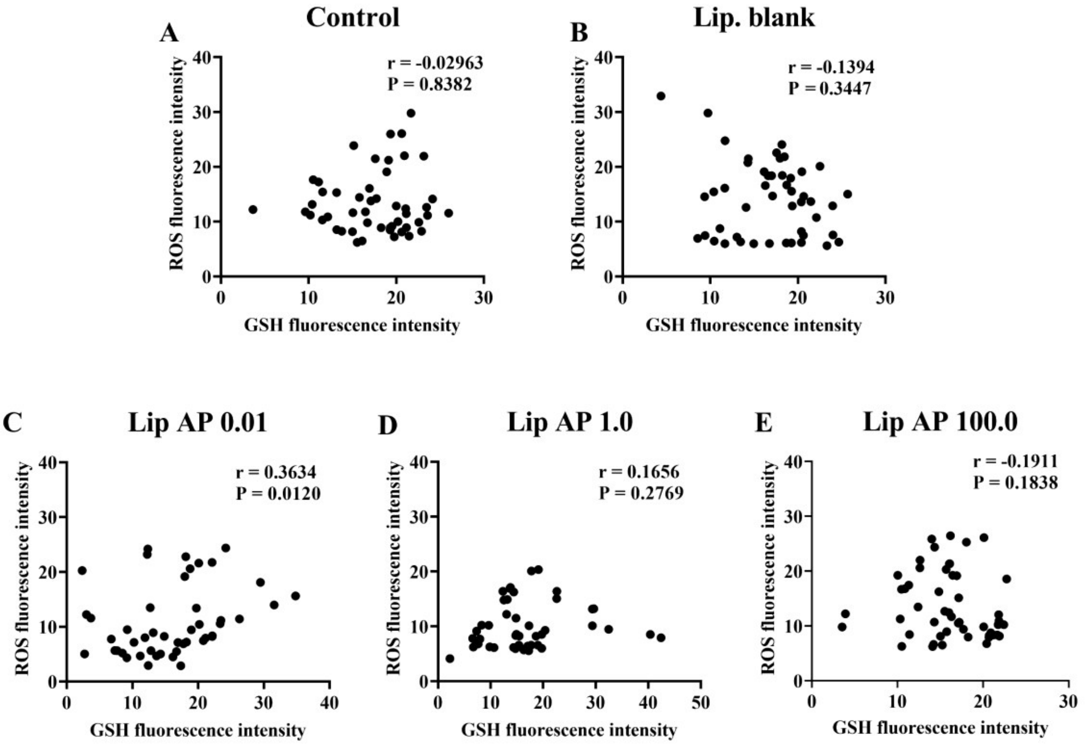

Figure_1_Supplnfo. Correlation between ROS and GSH levels in bovine oocytes matured in vitro in the control (n = 50), Lip-blank (n = 48) and Lip-α-pinene (AP) at 0.01 µg/mL (n = 47), 1.0 µg/mL (n = 45), and 100.0 µg/mL (n = 50). Each point represents one oocyte in eight repetitions of the experiment. Correlation analyses were performed using Spearman's correlation.
